# Preparedness of health facilities providing HIV services during COVID-19 pandemic and assessment of their compliance to COVID-19 prevention measures: findings from the Tanzania Service Provision Assessment (SPA) survey

**DOI:** 10.11604/pamj.supp.2020.37.1.25443

**Published:** 2020-09-30

**Authors:** Shraddha Bajaria, Ramadhani Abdul

**Affiliations:** 1 Ifakara Health Institute, Box 78373, Dar es Salaam, Tanzania,; 2 Independent Researcher, Shinyanga, Tanzania

**Keywords:** Tanzania, COVID-19, HIV, health system

## Abstract

**Introduction:**

the increased demands of health facilities and workers due to coronavirus overwhelm the already burdened Tanzanian health systems. This study evaluates the current capacity of facilities and providers for HIV care and treatment services and their preparedness to adhere to the national and global precaution guidelines for HIV service providers and patients.

**Methods:**

data for this study come from the latest available, Tanzania Service Provision Assessment survey 2014-15. Frequencies and percentages described the readiness and availability of HIV services and providers. Chi-square test compared the distribution of services by facility location and availability and readiness of precaution commodities and HIV services by managing authorities.

**Results:**

availability of latex gloves was high (83% at OPD and 95.3% laboratory). Availability of medical masks, alcohol-based hand rub and disinfectants was low. Availability of medical mask at outpatient department (OPD) was 28.7% urban (23.5% public; 33.8% private, p=0.02) and 13.5% rural (10.1% public; 25.4% private, p=0.001) and lower at laboratories. Fewer facilities in rural area (68.4%) had running water in OPD than urban (86.3%). Higher proportions of providers at public than private facilities in urban (82.8% versus 73.1%) and rural (88.2% versus 81.6%) areas provided HIV test counseling and at least two other HIV services.

**Conclusion:**

availability of commodities such as medical masks, alcohol-based hand rub, and disinfectant was low while the readiness of providers to multitask HIV related services was high. Urgent distribution and re-assessment of these supplies are necessary, to protect HIV patients, their caregivers, and health providers from COVID-19.

## Introduction

The emerge and spread of new coronavirus disease (COVID-19) globally has led to increased burden on health systems and workers [1]. While little evidence shows higher risk of COVID-19 among human immunodeficiency virus (HIV) patients than the general population, prevention measures in place can cause disruptions to HIV continuum of care [2]. In Tanzania, of the 1.6 million people living with HIV (PLHIV) in 2018, 1.4 million were on treatment [3]. Measures to control the pandemic could pose major challenges as PLHIV would not be able to or willing to visit health facilities to pick up medication [4-6]. Strict quarantine enforcement, limited means of regulated transport can limit patients´ can affect their ability to access health facilities; a study [4] in China found that 23% of PLHIV reported disruptions in uptake of medication while 68% reported worrying about disruption in medication and future clinical care. Another study [6] reported 32% of the PLHIV with risk of discontinuing their medication and almost half not knowing where to get their medications from. The requirement of wearing face mask while attending health facilities could discourage them due to costs of getting or making a mask. The patients might be unwilling to attend overcrowded facilities in fear of compromising their own health [7]. The pandemic could also pose major challenges in HIV monitoring services for newly diagnosed individuals and disruptions in supply and access of antiretroviral treatment (ART) drugs for those on treatment, ultimately leading to increased viral loads or deaths.

**COVID-19 situation in Tanzania:** the first case of COVID-19 in Tanzania was reported on March 16^th^ 2020 [8]. A rise in the number of cases to 299, by on April 28^th^ 2020, with 10 people deceased was reported [9]. The most recent record, accessed on 22^nd^ May 2020, shows 509 cases, with 21 fatalities. Like many other African countries, Tanzania has a young population, with more than half under the age of 25 years [10], who, according to published data, are at lower risk of contracting or dying of disease [11]. Tanzania is already struggling to build her health infrastructure and is burdened by diseases such as Malaria, HIV, tuberculosis (TB), and other non-communicable diseases (NCDs); therefore it is important to closely follow the COVID-19 situation. To contain the spread of COVID-19, the government banned all public gatherings, cancelled all in-person classes at schools and universities, suspended all political rallies and football league, recommended vising facilities only if symptomatic, avoid non-essential travel and over-crowded areas, within a few days of the first case being reported [12]. Restricted international travel and regulated travel to neighboring countries then followed, with mandatory 14 days self-quarantine for individuals entering the country [13]. However, a possibility of a total lockdown was ruled out [14] and places of worship remained open, encouraging citizens to attend prayers [15]. International travel restrictions were lifted on May 18^th^ 2020 and the airspace is now open, without the 14 days isolation requirement for people arriving to the country [16]. Students in their final year of secondary school and in universities also resumed in-class studies on June 1^st^ 2020 [17].

The national health authorities developed a guidance for healthcare workers (HCWs) and PLHIV working at and attending HIV Care and Treatment Clinics (CTCs) to maintain standard precautions against COVID-19 while providing essential HIV services to PLHIV [18]. Measures such as training on COVID-19 precautions and supervision during service provision, availability of personal protective equipment (PPE), utilization of telephones or virtual visits for non-urgent cases, encouraging workers to multitask, multi-month provision of antiretroviral (ARV) medication with phone follow up, mass masking of workers and clients with respiratory symptoms, prioritizing testing for those most in need, ensuring availability of ART and condoms, and counselling or creating awareness around COVID-19 best practices are recommended. The World Health Organization (WHO) also recommended infection prevention strategies such as use of masks [19] and proper water, sanitation and hygiene practices [20]. The President's Emergency Plan for AIDS Relief (PEPFAR) [21] also issued a technical guidance for PLHIV and service providers that recommends continuity of medication for PLHIV and adherence to infection prevention control guidelines. One study comparing the risk of infection among HCWs using a medical versus cloth mask reported higher risk among those using the latter [22]. Therefore, HCWs are recommended to use medical masks.

While the guidelines are necessary in protecting the PLHIV, HCWs and key populations from COVID-19, it is important to know the current status of the health system, level of preparedness, and see how to feasibly accommodate the new guidelines. A recent study [23] on infection prevention and control practices at outpatient facilities in Tanzania found inadequate compliance to the recommended practices; hand hygiene and disinfection of reusable equipment were especially rarely practiced by staff [23]. To understand the situation of HIV services, this study used the existing, publicly accessible data, the service provision assessment (SPA), which is a nationally representative survey that collects information on the overall availability of health services, appropriate infrastructure, resource availability, readiness of facilities and providers to deliver these services, whether standard guidelines are followed and client satisfaction [24]. Using the latest Tanzanian SPA (TSPA) data, this study aims to evaluate the health facilities´ preparedness for provision of HIV related services, HIV diagnostic, treatment and counseling services during the COVID-19 pandemic, and to understand how the precautions recommended for COVID-19 would be feasible given the current state of facilities providing HIV services. Specifically, the study will describe the current availability of HIV services and the readiness of providers to deliver these services, with a focus on the recommended COVID-19 precaution measures. The study results will highlight the barriers and facilitators of the facilities and HCWs, in their capacities of adhering to the interim guidelines.

## Methods

**Data source and management:** the latest TSPA survey was done in 2014-15. Using a multi-staged sampling method, 1200 health facilities from the country´s master list were surveyed. Further details are available in the report [25]. This study used facility and health provider data to identify HIV services available at the facilities and the preparedness of providers to deliver these services. Of the 1200 health facilities, 8 refused to participate in the survey and 4 were closed or non-functional; 1188 remaining facilities were used for this analysis. Of the 7015 health providers, only 6866 agreed to be interviewed.

**Details of measurement:** managing authority of health facilities was categorized into public and private, with parastatal considered public and mission/faith-based considered private. Categorization of regions as urban or rural was done in the questionnaire. Geographical regions from the mainland were also categorized into six zones, as per ministry of health classifications: Central, Coastal, Lake, Northern, Southern Highlands and Western [26]. The five regions from Zanzibar were all classified under the same category of ´Zanzibar´. Details of the facility and provider variables included in this study are presented below (Table 1). Readiness of providers to multitask was assessed using a derived variable; providers reporting to deliver at least three services, including counseling related to HIV testing were considered to have the ability to multitask. The availability of PMTCT services by facility managing authority was also included in this study.

**Table 1:** details of the facility and provider variables included in this study

Facility variables
**HIV testing services**
Capacity to conduct HIV testing, either by rapid diagnostic test or ELISA
An unexpired HIV rapid diagnostic test kit any other testing option is available in the facility the day of survey
**HIV care and support services**
treatment for any opportunistic infection or for symptoms related to HIV
Systematic intravenous treatment for specific fungal infections
Treatment of Kaposi's sarcoma
Palliative care such as symptom or pain management
Nutritional and supplementation services
Care for pediatric HIV patients
Preventive treatment for TB
Primary preventive treatment for opportunistic infections
Provision of condoms and family planning services
**HIV treatment services**
Prescription of ARV
Clinical follow up on clients on ARV
Outreach ARV facilities
**ARV medicines available**
Nucleoside reverse transcriptase inhibitors (NRTI)
Non-nucleoside reverse transcriptase inhibitors (NNRTI)
Protease inhibitors
Fusion inhibitor or combined ARVs
**Provider variables**
**HIV counseling or testing services**
Counseling related to HIV testing
Conducting HIV testing
Services related to prevention of mother to child transmission (PMTCT)
Palliative care services
Any ART services, including prescription, counseling and follow up
Preventive treatment for opportunistic infections
Pediatric AIDS care
Post-exposure prophylaxis (PEP)
**PMTCT services**
HIV testing and counseling
Infant feeding counseling
Nutritional counseling for HIV positive women and their infants
Family planning counseling for HIV positive women

**Statistical analysis:** data were cleaned and analyzed using Stata 15 software; frequencies and percentages described the readiness and availability of HIV services and providers. Chi-square test compared the distribution of HIV services by region and availability and readiness of HIV services by facility managing authorities. Bar graphs were used to present the distribution of HIV services by zones, distribution of specific PMTCT services by managing authority and proportions of facilities that reported to have COVID-19 precaution products available, by managing authority in urban and rural areas.

## Results

**Figure 1:**
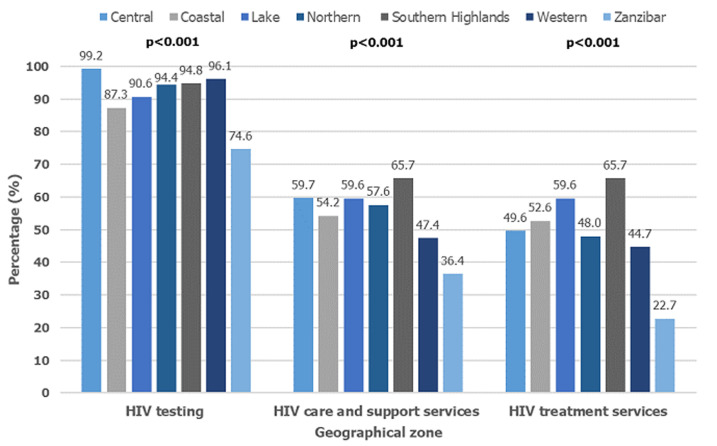
distribution of HIV services by geographical zones

**Availability of HIV services offered at facilities:** of the 1188 total facilities, 432 (36.4%; 49.3% public, 50.7% private) were located in urban area. Two thirds of all facilities 756 (63.6%) were in the rural areas, majority managed by the public 587 (77.7%). HIV testing was offered by 1081 (90.9%) facilities; 97.1% of all public and 78.4% of all private facilities. About one third of all facilities 347 (29.2%) did not provide any laboratory services in the facility. Only 618 (52.0% of all) facilities (56.1% public and 43.6% private) prescribed ARV treatment, and/or followed up clients on ARV). HIV care and support services were offered by 669 (56.3% of all) facilities; 61% of all publicly and 46.7% of all privately managed facilities. The distribution of HIV services by geographical zones are presented in Figure 1. There are disparities in the availability of HIV services by zones; Zanzibar has overall low availability in all aspects assessed. In general, HIV testing services are widely available in all zones with highest in Central zone, provided by over 99.2% of all facilities. Provision of HIV care and support services are at average 63.4%, with less than half of the facilities in Western zone (47.4%) reporting to provide HIV care and support services. In respect to HIV treatment services, Southern Highland zone was the highest (65.7%) followed by Lake and Coastal zones, 59.6% and 52.6% respectively, the remaining reported less than 50% (Figure 1).

**Figure 2:**
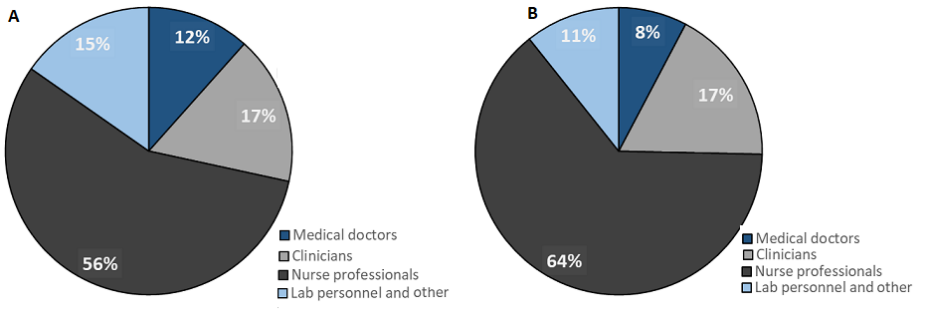
A) qualifications of health providers at private facilities; B) qualifications of health providers at public facilities

**Availability of health providers:** of the 6866 health providers, highest proportion 4200 (61.2%) were nurse professionals (57.7% of all providers in urban and 64.5% in rural areas), 1191 (17.4%) were clinicians, 627 (9.1%) were medical doctors (13% of all providers in urban and 5.4% in rural areas) and 848 (12.3%) were laboratory personnel or others. Almost three quarters (72.7%, 4991) of all providers reported providing any HIV counseling or testing service, of which, most 4732 (94.8%) provided counseling related to HIV testing. Of all the interviewed providers, about a quarter, 1590 (23.2%) reported to provide any laboratory service. Distribution of providers by qualification and managing authority is presented in Figure 2 A, B. Most of the interviewed providers worked at public facilities (63.6%), 36.5% worked at private facilities. The highest proportion of providers at both managing authorities were nurse professionals. Private facilities had slightly more medical doctors (12%) and laboratory personnel (15%) than public facilities (Figure 2).

**Figure 3:**
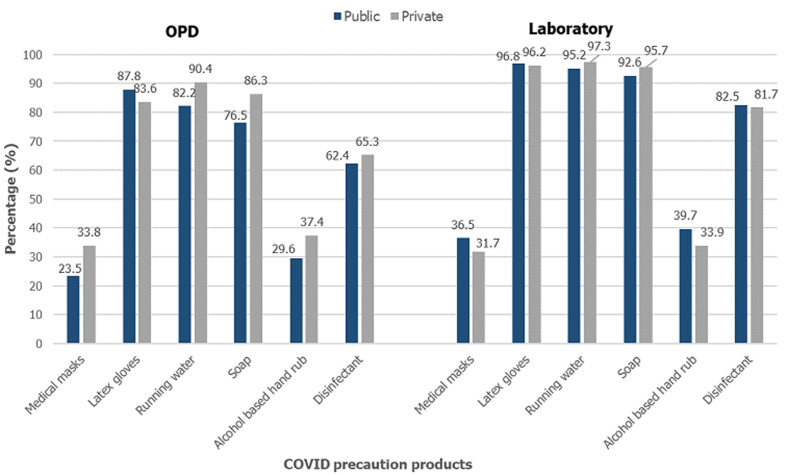
distribution of specific PMTCT services by facilities managing authority

**Availability of HIV PMTCT services and providers at facilities:** PMTCT services for HIV infection were provided at 995 (83%) facilities during antenatal care (ANC), of which 742 (74.6%) were public while 253 (25.4%) were privately managed. Distribution of specific PMTCT services is shown in Figure 3. Of the facilities that had PMTCT services, HIV testing and counseling was available at 97.7% of the public and 98.4% of the private facilities, infant feeding counseling at 717 (96.6%) public and 241 (95.3%) private facilities and nutritional counseling for women was done at most (95.2%) of the facilities, with no significant differences across managing authorities (p=0.682). Family planning counseling was done significantly more at public facilities 721 (97.2%) than private 216 (85.4%), p<0.001. Of all the interviewed health providers, 58.6% reported providing any PMTCT service, of which the highest proportion was provided by nurse professionals (77.3%), followed by clinicians (15.2%) and medical doctors (8.4%). Figure 3, Table 2 and Table 3 describe HIV services availability and providers readiness by taking into consideration recommendations from Tanzania COVID-19 prevention guidelines for HIV service sites. Annex 1 presents the total frequencies of facilities and providers by service type, managing authority and facility location, to aide in the interpretation of Table 1 and Table 2.

**Table 2:** proportions of available COVID-19 prevention commodities and HIV services at urban facilities by managing authority

	Overall		Public		Private		p-value
	**n**	**(%)**	**n**	**(%)**	**n**	**(%)**	
**Availability indicators**							
**Availability of protective gear**							
**General OPD area (n=432)**							
Medical masks	124	(28.7)	50	(23.5)	74	(33.8)	0.018
Gloves	370	(85.7)	187	(87.8)	183	(83.6)	0.210
**Laboratory (n=375)**							
Medical masks	128	(34.1)	69	(36.5)	59	(31.7)	0.328
Gloves	362	(96.5)	183	(96.8)	179	(96.2)	0.755
**Availability of sanitation products**							
**General OPD area (n=432)**							
Running water	373	(86.3)	175	(82.2)	198	(90.4)	0.013
Soap	352	(81.5)	163	(76.5)	189	(86.3)	0.009
Alcohol based hand-rub	145	(33.6)	63	(29.6)	82	(37.4)	0.083
Disinfectant	276	(63.9)	133	(62.4)	143	(65.3)	0.537
**Laboratory (n=375)**							
Running water	361	(96.3)	180	(95.2)	181	(97.3)	0.290
Soap	353	(94.1)	175	(92.6)	178	(95.7)	0.201
Alcohol based hand-rub	138	(36.8)	75	(39.7)	63	(33.9)	0.243
Disinfectant	308	(82.1)	156	(82.5)	152	(81.7)	0.836
**Availability of any ARV drug (n=241)**							
Any NRTI	166	(68.9)	124	(72.5)	42	(60.0)	0.057
Any NNRTI	218	(90.5)	158	(92.4)	60	(85.7)	0.109
Any protease inhibitors	88	(36.5)	65	(38.0)	23	(32.9)	0.451
Any combination ARV drug	224	(92.9)	162	(94.7)	62	(88.6)	0.090
**Availability of condoms**							
HIV testing site **(n=351)**	234	(66.7)	163	(79.5)	71	(48.6)	0.000
HIV care and support site **(n=242)**	183	(75.6)	146	(86.9)	37	(50.0)	0.000
**Readiness indicators**							
**Readiness of facility for any functioning communication system (n=432)**	277	(64.0)	128	(60.1)	149	(68.0)	0.085
Functioning landline	157	(36.3)	69	(32.4)	88	(40.2)	0.092
Functioning cellphone	230	(53.2)	111	(52.1)	119	(54.3)	0.643
Functioning computer	282	(65.3)	155	(72.8)	127	(57.9)	0.001
Access to email	215	(49.8)	111	(52.1)	104	(47.5)	0.337
**Readiness of providers to multitask (n=2124)**							0.000
HIV test counseling and 1 other service	324	(15.3)	216	(14.3)	108	(17.7)	
HIV test counseling and 2 other services	548	(25.8)	392	(25.9)	156	(25.6)	
HIV test counseling and 3 or more other services	1151	(54.2)	862	(56.9)	289	(47.5)	
**Any HIV laboratory service (n=763)**	659	(86.4)	412	(95.2)	247	(74.9)	0.000

**Table 3:** proportions of available COVID-19 prevention commodities and HIV services at rural facilities by managing authority

	Overall		Public		Private		p-value
	**n**	**(%)**	**n**	**(%)**	**n**	**(%)**	
**Availability indicators**							
**Availability of protective gear**							
**General OPD area (n=756)**							
Medical masks	102	(13.5)	59	(10.1)	43	(25.4)	0.000
Gloves	604	(79.9)	465	(79.2)	139	(82.3)	0.386
**Laboratory (n=466)**							
Medical masks	115	(24.7)	54	(16.9)	61	(41.8)	0.000
Gloves	443	(95.1)	303	(94.7)	140	(95.9)	0.578
**Availability of sanitation products**							
**General OPD area (n=756)**							
Running water	517	(68.4)	363	(61.8)	154	(91.1)	0.000
Soap	498	(65.9)	351	(59.8)	147	(86.9)	0.000
Alcohol based hand-rub	174	(23.0)	110	(18.7)	64	(37.9)	0.000
Disinfectant	432	(57.1)	323	(55.0)	109	(64.5)	0.028
**Laboratory (n=466)**							
Running water	406	(87.1)	267	(83.4)	139	(95.2)	0.000
Soap	396	(84.9)	260	(81.3)	136	(93.2)	0.001
Alcohol based hand-rub	167	(35.8)	94	(29.4)	73	(50.0)	0.000
Disinfectant	350	(75.1)	228	(71.3)	122	(83.6)	0.004
**Availability of any ARV drug (n=377)**							
Any NRTI	171	(45.4)	110	(39.6)	61	(61.6)	0.000
Any NNRTI	341	(90.5)	247	(88.9)	94	(94.9)	0.076
Any protease inhibitors	65	(17.2)	34	(12.2)	31	(31.3)	0.000
Any combination ARV drug	348	(92.3)	254	(91.4)	94	(94.9)	0.251
**Availability of condoms**							
HIV testing site **(n=730)**	463	(63.4)	404	(70.6)	59	(37.3)	0.000
HIV care and support site **(n=427)**	290	(67.9)	246	(76.9)	44	(41.1)	0.000
**Readiness indicators**							
**Readiness of facility for any functioning communication system (n=756)**	249	(32.9)	152	(25.9)	97	(57.4)	0.000
Functioning landline	39	(5.2)	9	(1.53)	30	(17.8)	0.000
Functioning cellphone	226	(29.9)	139	(23.7)	87	(51.5)	0.000
Functioning computer	262	(34.7)	152	(25.9)	110	(65.1)	0.000
Access to email	150	(19.8)	68	(11.6)	82	(48.5)	0.000
**Readiness of providers to multitask (n=2608)**							0.000
HIV test counseling and 1 other service	281	(10.8)	180	(10.0)	101	(12.4)	
HIV test counseling and 2 other services	850	(32.6)	627	(34.9)	223	(27.5)	
HIV test counseling and 3 or more other services	1397	(53.6)	958	(53.3)	439	(54.1)	
**Any HIV laboratory service (n=827)**	779	(94.2)	556	(95.2)	223	(91.8)	0.054

**Figure 4:**
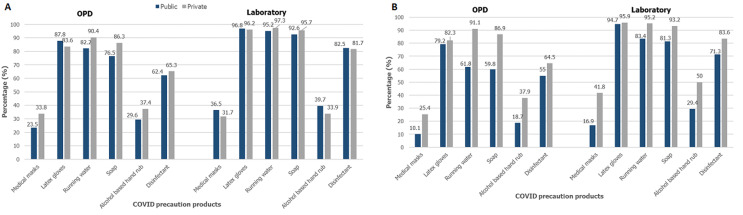
A) availability of COVID-19 prevention products at facilities in urban area; B) availability of COVID-19 prevention products at facilities in rural area

**Availability of protective gear, sanitation products, ARV medication and condoms in urban area:** availability of medical masks in the facilities in general outpatient department (OPD) area was generally low 124 (28.7%), higher in facilities managed by private 74 (33.8%) than public 50 (23.5%), also shown in Figure 4 A, the difference was significant at p=0.018. Only 34.1% of the laboratories surveyed had masks available; there were no differences between public and privately managed facilities. Availability of latex gloves was universal, majority of the facilities reported availability of gloves at general OPD (85.7%) and laboratories (96.5%). Running water (p=0.013) and soap (p=0.009) were available significantly more at OPD of private facilities than public. Alcohol-based hand rub and disinfectant were available at only 33.6% and 63.9% respectively, of all facilities, with non-significant differences across managing authorities. More than three quarters of all laboratories had running water (96.3%), soap (94.1%) and disinfectant (82.1%) available, only 36.8% had alcohol-based hand rub, with no significant difference between public and private facilities, as shown in Figure 4 A, B, Table 2 and Table 3. Large proportion of facilities reported having any NRTI (68.9%), any NNRTI (90.5%) and any combination ARV drug (92.9%), while only 36.5% had any protease inhibitors, the difference was non-significant across managing authorities. Of all the facilities that provided HIV testing services, 234 (66.7%) reported to have condoms available for clients; significantly higher in public facilities than private (79.5% versus 48.6%; p<0.001), the same trend was also observed in the facilities that reported having HIV care and support services available; 146 (86.9%) public and 37 (50.0%) private facilities reported availability of condoms (p<0.001).

**Availability of protective gear, sanitation products, ARV medication and condoms in rural area:** availability of medical masks was low 102 (13.5%) overall at OPD areas, with private facilities 43 (25.4%) significantly more than at public facilities 59 (10.1%), p<0.001 (Figure 4 B). Only 24.7% of laboratories had masks available with significantly more at private facilities 61 (41.8%) compared to public 54 (16.9%), p<0.001. More than three quarters (80%) of OPD of all facilities and 95% of all laboratories had latex gloves, with no significant differences across managing authorities. As shown in Figure 4 B, availability of all sanitation products in OPD areas was higher at private facilities than public (running water, soap, alcohol based hand rub p<0.001, disinfectant p=0.028). Alcohol based hand rub was only available at OPD of 23.0% of all (18.7% of public and 37.9% of private) facilities. Running water (p<0.001) and soap (p=0.001) were available significantly more at laboratories of public than private facilities. Alcohol based hand rub was available at 50.0% of private and 29.4% of public facilities, p<0.001. Disinfectant was generally available at 350 (75.1%) laboratories, significantly higher at private than public facilities (p=0.004). Any NRTI (p<0.001) and any protease inhibitors were available at significantly more private facilities than public. Majority of facilities reported having any NNRTI (90.5%) and any combination drug (92.2%), with no significant differences across managing authorities. Two thirds of both, HIV testing sites and HIV care and support sites had condoms available, with public facilities having significantly more than private (p<0.001).

**Readiness of facilities for COVID-19 precaution measures in urban area:** overall, 64% of the facilities had any functioning communication system, with no significant difference across managing authorities. Higher number of public facilities had functioning computer than private (155 (72.8%) versus 127 (57.9%); p=0.001), only about half of facilities 230 (53.2%) reported having a functional cellphone (111 (52.1%) for public and 119 (54.3%) for private; p=0.643). Access to emails were moderate at 215 (49.8%), slightly higher in the public facility 111 (52.1%) than private 104 (47.5%). In respect to facility readiness to provide multiple HIV services under one roof as per recommendation, majority of the providers reported providing counseling related to HIV testing and two or more other services. Specifically, a quarter of providers at both public and private facilities reported to provide counseling related to HIV testing and two other services, and more providers at public facilities (56.9%) reported to provide counseling related to HIV testing and three or more other services compared to private (47.5%). A small proportion (4.8%) of all providers reported providing only counseling related to HIV testing in their current positions, with more at private facilities (9.2%) than public (2.9%) (Table 2).

**Readiness of facilities for COVID-19 precaution measures in rural area:** significantly more private than public facilities had functioning communication systems than (p<0.001), with 30 (17.8%) private facilities having a functioning landline, compared to 9 (1.5%) public facilities, and more than half of private facilities 87 (51.5%) with a functioning cellphone, compared to 139 (23.7%) of public facilities. Providers at public facilities were more likely to provide multiple HIV services, i.e. counseling related to HIV testing and two other services (34.9%), compared to private facilities (27.5%), p<0.001. Slightly more providers at private facilities were able to provide counseling related to HIV testing and three or more other services (54.1%) compared to public (53.3%). Very few providers 80 (3.1%) reported providing only counseling related to HIV testing in their current positions (Table 3).

## Discussion

The findings from this study show limited availability of some COVID-19 precaution products, such as medical masks, disinfectants and alcohol based hand rub while the readiness of facility providers to multitask HIV related services was fairly high. The results have also revealed unequal access to running water supply between urban and rural as well as between private and public facilities, access to running water in rural facilities was generally low. Availability of medical masks at facilities in general was considerably low, especially at publicly managed facilities and facilities in rural areas. The use of medical masks has been recommended by the Tanzanian government as well as the WHO in the interim guidance for rational use of PPE [27] and the advice on use of mask in context of COVID-19 [19]. This shortage could put the limited health providers at high risk of being infected and spreading the infection to clients, especially immunosuppressed HIV clients. The WHO also called on industries and governments to increase production of PPE, since the globally increased demand and prices as well as disruptions in import-export of supplies could lead to severe shortages [28]. Latex gloves were largely available at facilities, with no significant differences across managing authorities, and slightly more available at laboratories of facilities in both, urban and rural areas. This finding is reflected in a recent study that indicates high compliance of HCWs at private facilities using gloves, especially when carrying out intravenous injection, blood draw, wound cleaning or dressing and using new gloves for each patient [23].

Overall, the availability of running water was insufficient, especially at OPD areas of public facilities in rural regions. The WASH and waste management interim guideline by WHO emphasize frequent and correct hand hygiene to prevent human-to-human transmission of COVID-19 [20]. Without running water, following this basic prevention measure would be hindered [1]. Availability of soap was moderate to high, with higher proportions of laboratories of privately managed facilities reporting to have it and very low availability at OPD areas of public facilities in rural regions. Results from a study in China showed an increased risk of infection among HCWs was linked to suboptimal hand hygiene practice [29]. Alcohol based hand rub was the least available sanitation product in general, with less than half of all (OPD and laboratories) facilities in urban and rural areas having it available. The differences across public and privately managed facilities were statistically significant in rural areas, with more privately managed facilities having alcohol based hand rub available, especially at laboratories. Despite that the data used for the current study were collected five years ago, the findings are consistent with results from a recent study [23] which shows only 12% of the outpatient consultation rooms of private facilities had adequate hand hygiene facilities, including water, soap, single-use hand towels and gel sanitizers, and the practice was rarely adhered to by HCWs. Similarities between the two studies reveal slow pace in improvement in availability of some essential items at Tanzanian facilities in the past five years. Our findings indicate that availability of disinfectant was also generally low, with significantly lower at OPD of public facilities in rural area compared to private (55.0% public versus 64.5% private facilities, p=0.028). In contrast to this, disinfection of reusable equipment was the least adhered to guideline for infection control by HCWs at private OPD in Tanzania [23]. The study [23] included clinical observations for disinfection of common reusable equipment such as thermometers, stethoscopes and otoscopes, before and after attending to each client; only 13% of thermometers and less than 1% of stethoscopes were disinfected between patient visits [23]. The results from current study compared to the previous study indicate that although a product can be available, the readiness of providers to follow the guidelines requires training and supervision.

Ensuring adequate supply of medication, through multi-month dispensing of ARV medications, ideally for 30 days or more if possible [11] is highly recommended. This would prevent stable patients from visiting health facilities frequently to pick up medication and being potentially exposed to infection. However, this would only be possible if health facilities have enough medication in stock to provide clients with more medication than usual. In this study, most of the facilities reported having combination ARV drug available hence more likely to adhere to this recommendation, however further studies should assess the magnitude of the stock availability in case of multi-month prescription. Telephone or virtual visits have also been recommended for non-urgent care and counseling related to HIV, in order to minimize the number of patient visits to health facilities and reduce their risk of COVID-19 infection. Overall, moderate (64.0% in urban and 32.9% in rural area) proportions of facilities reported having a functioning means of communication; significantly more at private facilities in rural area than public. Due to the moderate availability, this recommendation can only be followed by about half of all facilities and even more difficult to be implemented at public facilities in the rural area. Instead of depending on facility phones, health providers can use their personal mobile phones to follow up on HIV patients, since mobile use penetration is growing at 85% penetration in 2020 [10]. Previous studies have indicated positive outcomes through use of mobile technology by village HCWs for case management of malaria [30], as well as delivering information and promoting family planning through mobile texting [31]. To reduce the number of interactions HIV patients have during a visit to health facility, the national interim guidelines and the PEPFAR technical guidance [18,21] recommend that staff multitask, as such, results from this study indicate that about a quarter of all health providers at facilities in urban area provide counseling related to HIV testing and two other services compared to about one third at facilities in rural area; approximately half of all providers reported providing counseling related to HIV testing and three or more other HIV related services, the combined proportions were slightly higher for providers at facilities in rural area, than urban. Although this could be due to shortages and uneven distribution of HCWs in rural facilities [32], their reported provision of multiple HIV services shows a major strength in context of preparedness of providers for COVID-19 response.

**Strengths and limitations:** although data were collected five years ago, the strength lies in the national-level representativeness of facilities and providers, and are the latest service provision data publicly available. Recent study done in private facilities has shown similar level of availability of water, disinfectant and hand rub, which indicates no substantial improvement in some areas, hence increasing the reliability of the findings from this study. To the authors´ knowledge there is no other publicly available, nationally representative data that includes all types of health facilities and management authorities. This study is not without limitations; the data were collected five years ago and it could be argued that the findings will not reflect the most current situation in some areas, availability of any most recent national representative survey would have increased the strength of this study. Additionally, this study did not take into consideration the facility level or size, availability or readiness of some items at the facility would have varied by facility level or size; future studies can analytically account for these.

## Conclusion

These findings highlight the challenges in implementing COVID-19 precaution measures and suggest a need to urgently make all sanitation and hygiene products available at facilities and ensure an adequate supply of medical masks especially in rural areas, to adhere to the national interim guidelines for HIV service provision and protect the PLHIV, their caregivers and health providers from COVID-19. The findings also indicate the ability of providers to multitask HIV services, thus reduce the number of interactions HIV patients would have during a visit, conforming to the COVID-19 guidelines. The study outcomes are also relevant for other low-income countries that have similar service provision settings as Tanzania.

### What is known about this topic

- The preparedness of health facilities in Tanzania to provide HIV services is well documented in multiple studies and surveys done previously;
- Prevention measures such as physical and social distancing, isolation, sanitation and frequent hand hygiene practices are recommended for the novel COVID-19.

### What this study adds

- The preparedness of health facilities in Tanzania to comply to COVID-19 prevention measures while providing HIV-services is low, and could possibly overwhelm the already burdened health system;
- There is an urgent need of reallocating resources and increasing availability of sanitation and hygiene products at facilities in order to protect the HIV patients.

## Annex

**Annex 1**: frequencies of facilities and providers by specific service, managing authority and facility location (PDF-205 KB)
